# Compensated Row-Column Ultrasound Imaging System Using Multilayered Edge Guided Stochastically Fully Connected Random Fields

**DOI:** 10.1038/s41598-017-09534-1

**Published:** 2017-09-06

**Authors:** Ibrahim Ben Daya, Albert I. H. Chen, Mohammad Javad Shafiee, Alexander Wong, John T. W. Yeow

**Affiliations:** 1 Vision and Image Processing (VIP) Research Group, Department of Systems Design Engineering, University of Waterloo, 200 University Avenue W, Waterloo, ON N2T 3G1 Canada; 2 Advanced Micro-/Nano- Devices Research Group, Department of Systems Design Engineering, University of Waterloo, 200 University Avenue W, Waterloo, ON N2T 3G1 Canada

## Abstract

The row-column method received a lot of attention for 3-D ultrasound imaging. By reducing the number of connections required to address the 2-D array and therefore reducing the amount of data to handle, this addressing method allows for real time 3-D imaging. Row-column still has its limitations: the issues of sparsity, speckle noise inherent to ultrasound, the spatially varying point spread function, and the ghosting artifacts inherent to the row-column method must all be taken into account when building a reconstruction framework. In this research, we build on a previously published system and propose an edge-guided, compensated row-column ultrasound imaging system that incorporates multilayered edge-guided stochastically fully connected conditional random fields to address the limitations of the row-column method. Tests carried out on simulated and real row-column ultrasound images show the effectiveness of our proposed system over other published systems. Visual assessment show our proposed system’s potential at preserving edges and reducing speckle. Quantitative analysis shows that our proposed system outperforms previously published systems when evaluated with metrics such as Peak Signal-to-Noise Ratio, Coefficient of Correlation, and Effective Number of Looks. These results show the potential of our proposed system as an effective tool for enhancing 3-D row-column imaging.

## Introduction

Ultrasound imaging is a valuable tool in non destructive testing, with applications ranging from detection of material defects to object and foreign body detection^1^. 3-D ultrasound imaging offers the possibility of accurately generating certain material properties that could be useful to material scientists^2^. 3-D ultrasound imaging could also be useful in medical imaging: it is difficult to image the same slice in 2-D for the purpose of follow up studies, and viewing of anatomy using a 2-D imaging device requires a great deal of skill and experience^3^.

For 3-D ultrasound imaging systems, the use of a fixed transducer with electronic beam-steering is preferred over a mechanically moving transducer, as mechanical motion introduces unwanted artifacts and the increased acquisition time does not allow for applications requiring real time feedback. A 2-D array of transducers is needed to achieve 3-D ultrasound imaging without mechanical motion. However, a fully addressed *N* × *N* 2-D requires *N* ^2^ connections, which offers a challenge both in addressing individual connections as well as acquiring and processing large amounts of data^4^. One way to address this issue that has received a great deal of attention in literature is to use the row-column method^4–8^.

**Figure 1:**
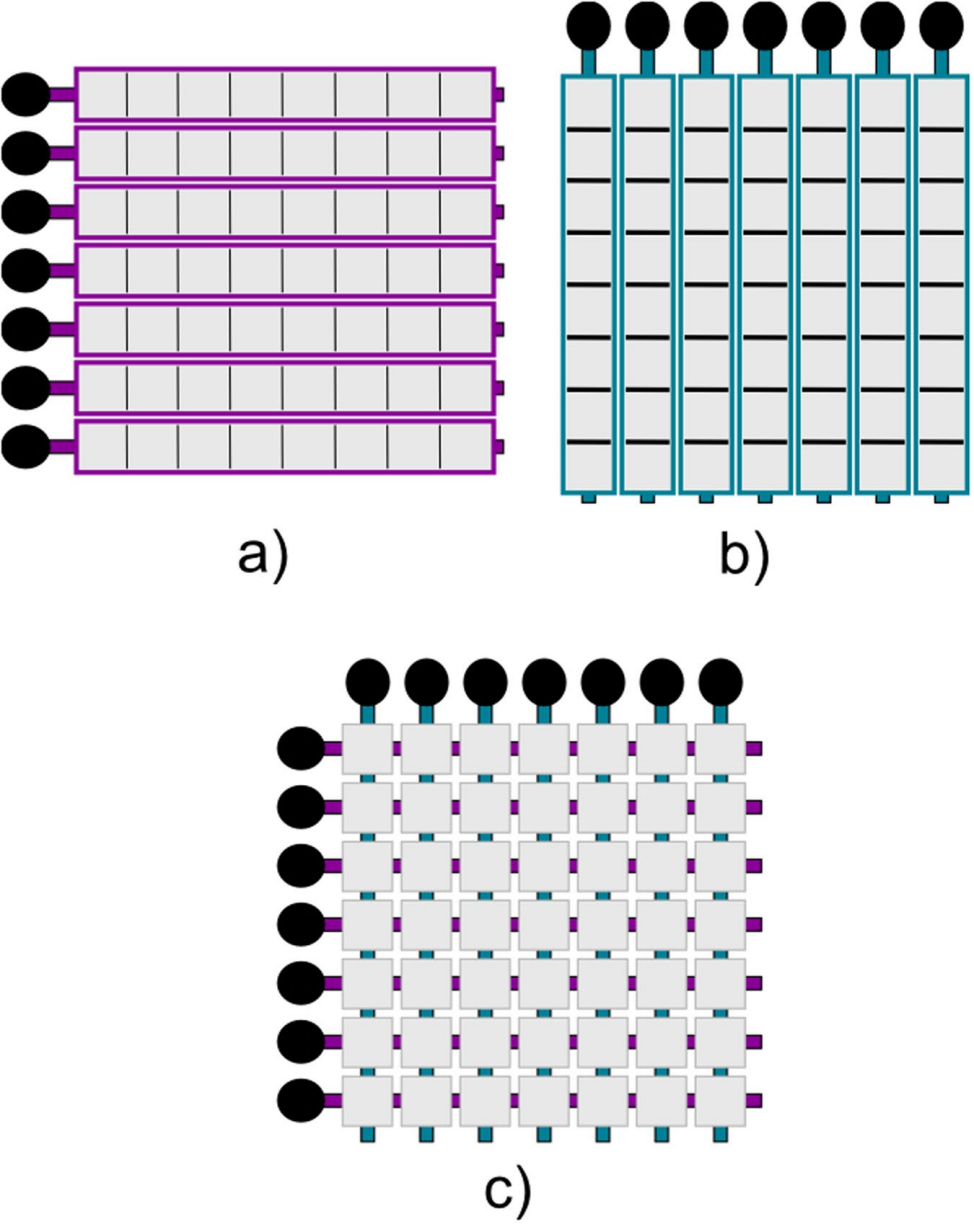
Visualization of a row-column array. (**a**) shows *N* row arrays with *N* connections, (**b**) shows *N* column arrays with *N* connections, (**c**) shows the row-column arrangement with 2*N* connections.

Proposed by ref. 5, the row-column method suggests the use of a pair of orthogonally positioned 1-D arrays of rows and columns, where one is responsible for transmit beamforming and the other for receive beamforming. A line of focus, adjustable in both depth and azimuth, is generated in a manner similar to 1-D transmit beamforming by the column array. Receive beamforming is achieved when the sound reflected from the object being imaged is received by the row array. Using this 2-D transducer setup, the number of connections required is only 2*N* instead of *N* ^2^ ^9^. A visualization of the row-column method is shown in Fig. 1.

**Figure 2:**
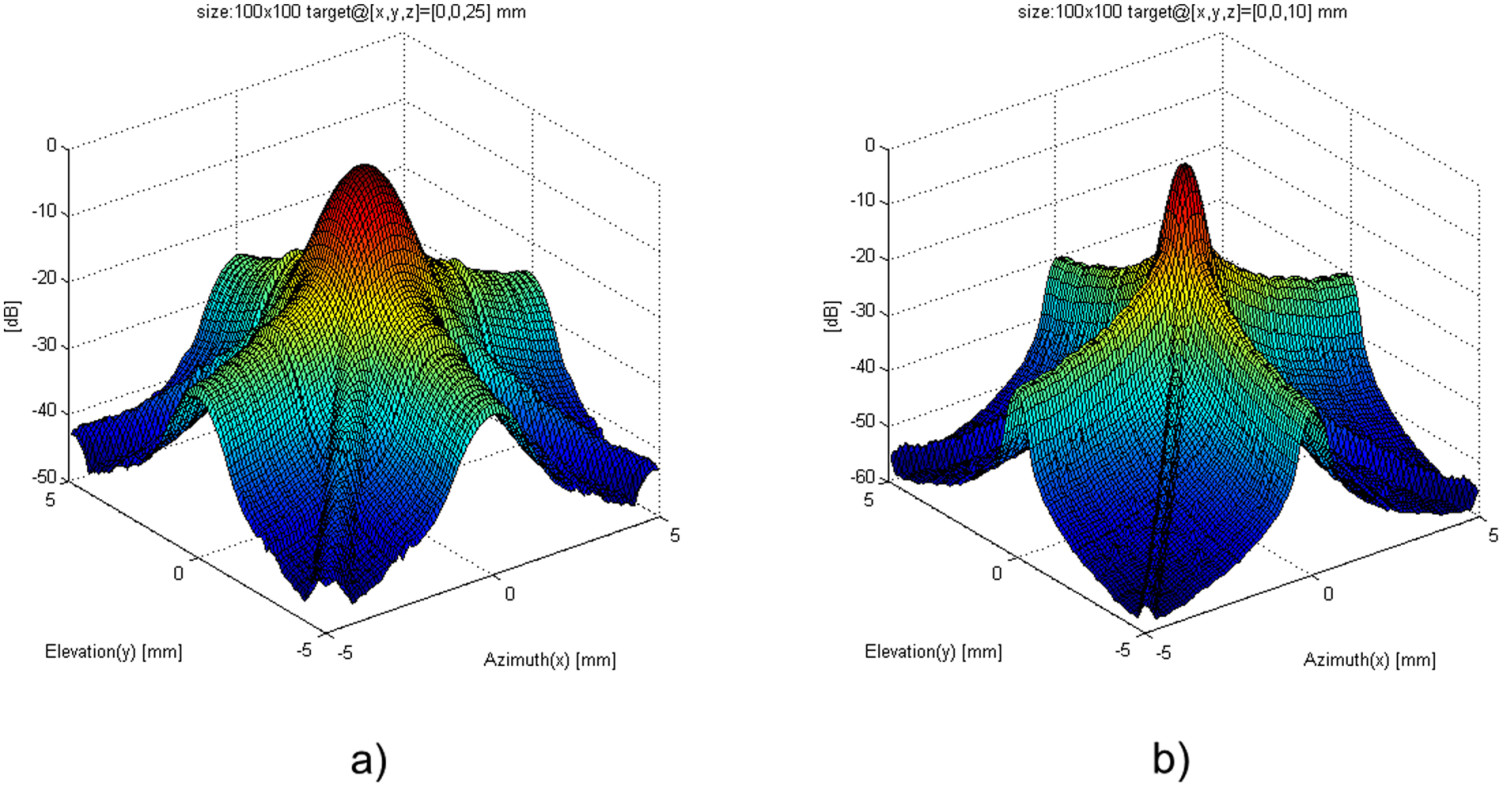
The point spread function of a 5 mm × 5 mm, 32 × 32 elements row-column array. The −6 dB resolution weakens as the focusing and scatterer moves from 10 mm (**a**) to 25 mm (**b**) away from the aperture. Side lobes can be seen below −30 dB. The two plots were generated by scanning an ideal point target located at [x, y, z] = [0, 0, 10] mm and [0, 0, 25] mm. The maximum value of the demodulated received beamformed signal was plotted as a function of the lateral distance in the azimuth and elevation direction 20 mm away from the aperture. The demodulated signals are normalized and presented with an 80 dB dynamic range.

There are some intrinsic limitations to the row-column method. For ultrasound waves, sound pressure emitted from the transducers gradually weakens as it moves away, causing a beam profile that varies the response of the imaging system with depth. This means that the point spread function (PSF) of ultrasound systems is spatially dependant (as demonstrated in Fig. 2), and must be considered as such in a proper reconstruction framework. The row-column’s PSF also suffers from ghosting artifacts caused by edge waves^4^, which degrades the reconstructed image. Ultrasound imaging in general also suffers from data sparsity and speckle noise inherent to ultrasound. All these challenges cannot be addressed by sensor design alone, a reconstruction framework must be proposed that can take all these challenges into account.

A number of row-column systems were proposed in literature. A system proposed by Chen *et al*.^6^ achieves real-time ultrasound imaging by incorporating a capacitive micromachined ultrasound transducers (CMUTs) based row-column addressing scheme. However, this system poorly addresses data sparsity through bi-linear interpolation and does not account for speckle noise or the spatially-varying PSF. Another system proposed by Rasmussen *et al*.^4^ and Christiansen *et al*.^7^ attempts to directly address some of the limitations of row column by introducing line beamforming and using integrated apodization instead of Hann apodization to improve image quality, it still does not take speckle noise into account. A recent row-column system proposed by Ben Daya *et al*.^10^ incorporated a reconstruction framework that compensates for the intrinsic limitations of row-column; using multilayered conditional random fields (MCRF) to account for data sparsity, speckle noise, and a spatially varying PSF with inherent ghosting artifacts has shown promising results. However, the use of MCRF leads to over-smoothed images with poorly preserved edges, and the way they account for the ghosting artifacts has not been properly addressed. In this research, we propose an edge-guided compensated row column ultrasound imaging system (henceforth referred to as EG-CRCUIS) that builds on the row-column ultrasound imaging system proposed in ref. 10 by incorporating a multilayered edge-guided, stochastically-fully connected random fields (MEG-SFCRF) to address the limitations of that system.

The rest of the document is organized as follows: we will first present the main stages of the EG-CRCUIS in the method section. Next, we will outline our experimental setup to test our new method. We will then show the results of our experiments and discuss them. Finally we will conclude with a summary and propose future work.

## Method

**Figure 3:**
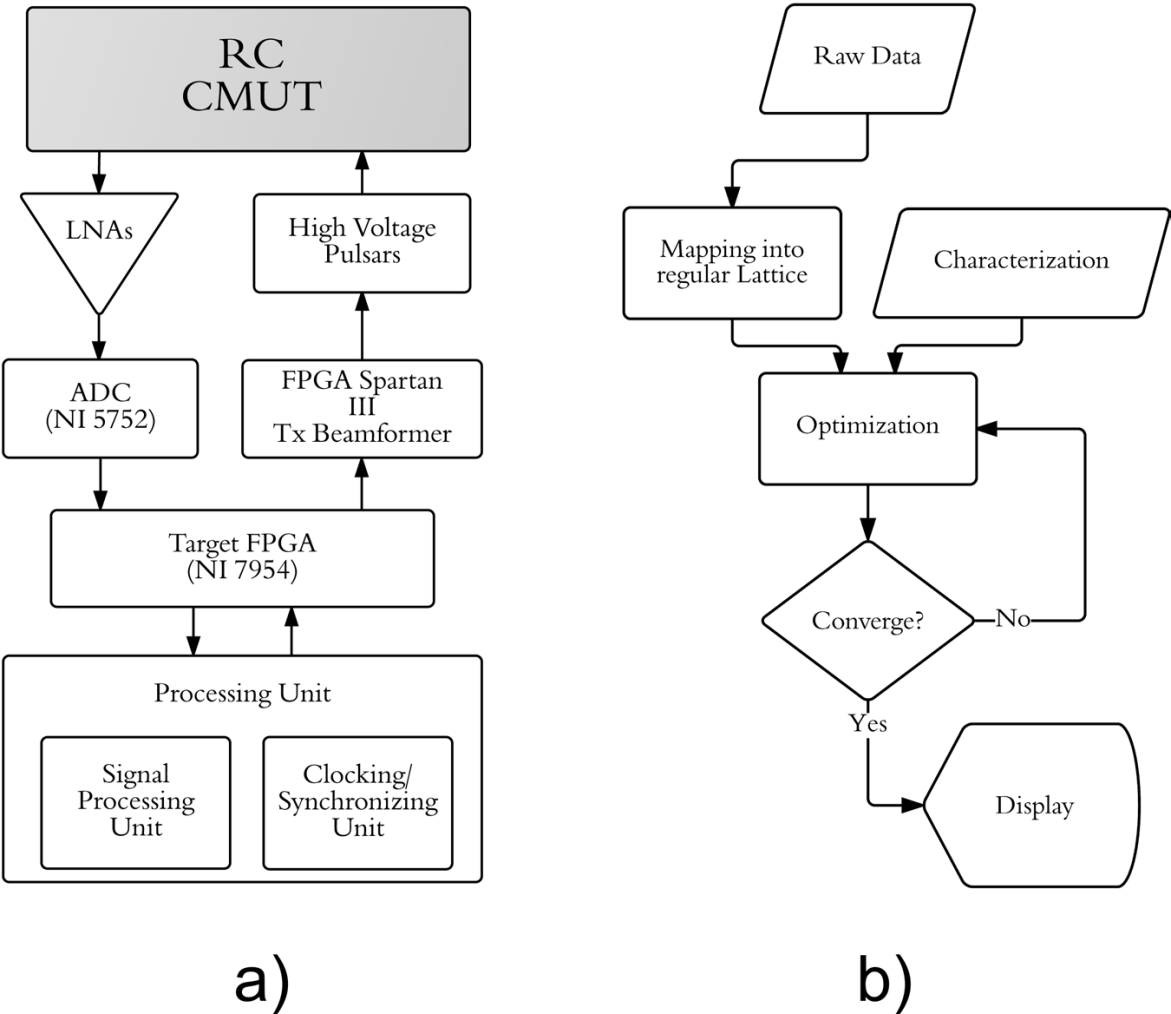
A flow chart representing the proposed system. (**a**) Shows the a top level implementation of the EG-CRCUIS. (**b**) Shows the signal processing unit in more detail.

The compensated row-column ultrasound imaging system (CRC-UIS) proposed in ref. 10 contains three main stages: data acquisition, characterization, and signal processing. Our main contribution is in the signal processing stage, where we incorporate the MEG-SFCRF model. A top level implementation of system can be seen in Fig. 3.

### Data Acquisition

The unit’s block diagram - shown in Fig. 3 - consists of a customized system built using the PCI eXtensions for Instrumentation (PXI) platform. A row-column addressing capacitive micromachined ultrasonic transducers array (RC-CMUTs) was used, with pre-amplifiers being added to compensate for its small current output signal. A digitizer, a field-programmable gate array (FPGA) board, and an embedded controller module are also included. An external FPGA was included for transmit beamforming, as well as a set of high voltage pulsers for stepping up voltage^10^.

### Characterization

This subsection details the characterization of the intrinsic properties of the data acquisition unit, which will be used as input to the signal processing unit. First we discuss the mathematics of how an image is observed, then we present how noise is modelled, finally we describe how the PSF is characterized at different depths.

#### Image Formation

Equation 1 describes how a true image is observed when the row-column technique is used:

1 $${g}_{r}(x,y,z)=M(x,y,z)\,[f(x,y,z)\ast h(x,y,z)+u(x,y,z)].$$

where *x*, *y*, and *z* are the Cartesian coordinates. The term *g*_*r*_(*x*, *y*, *z*) is the observed RF image, *M*(*x*, *y*, *z*) is the sampling function, *f*(*x*, *y*, *z*) encodes the tissue reflectivity function, the operator ‘*’ denotes the convolution operation, *h*(*x*, *y*, *z*) represents the spatially dependent point spread function (PSF); a function that describes the response of an imaging system to a point source, and *u*(*x*, *y*, *z*) is the noise component^11^.

**Figure 4:**
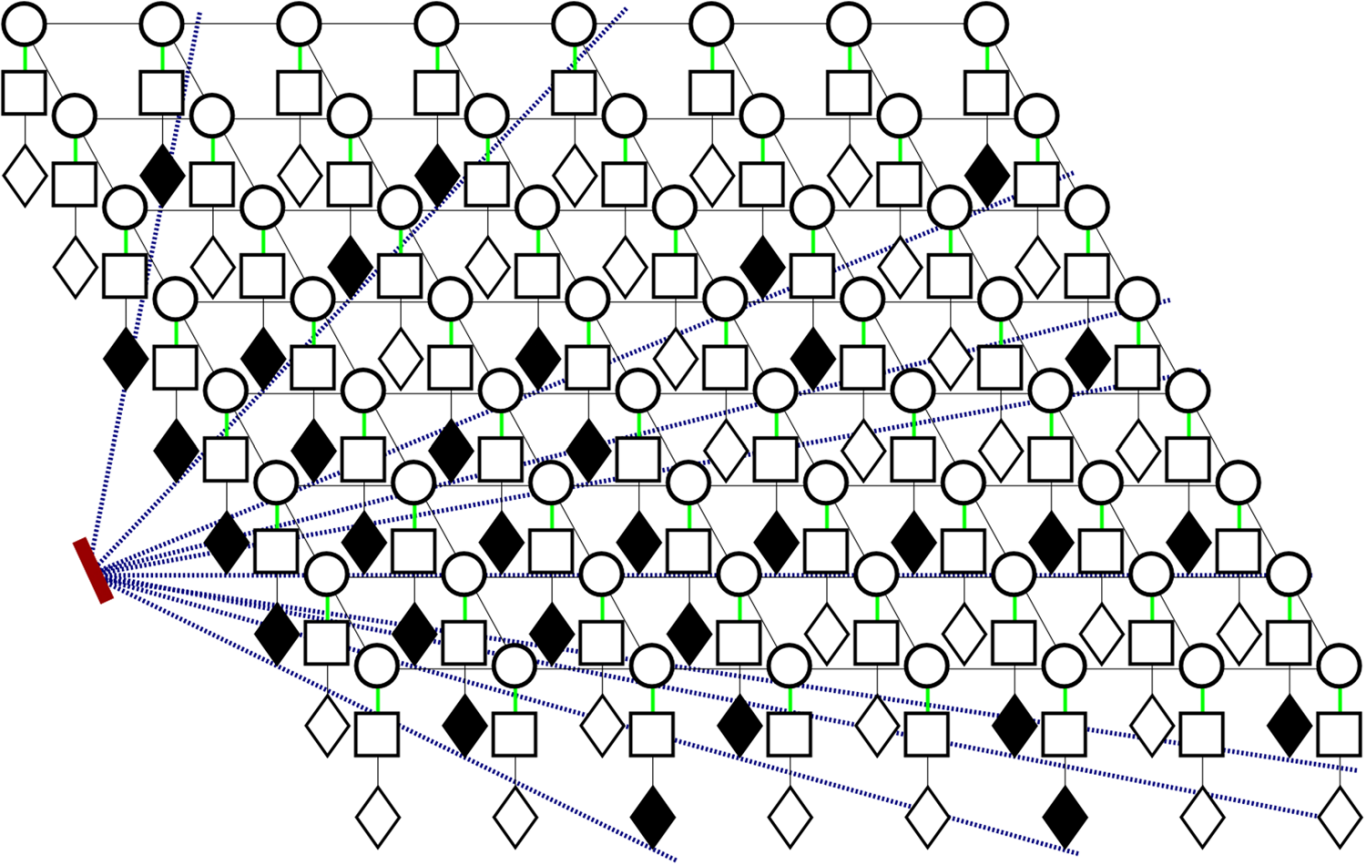
Fan beams originating from the ultrasound transducer. Black diamonds indicate available readings, white diamonds indicate absent readings that need to be estimated.

The observed RF image *g*_*r*_(*x*, *y*, *z*) is a series of fan-beams of ‘readings’, originating from the ultrasound source, in a three dimensional black box. This is visualized in Fig. 4. The sampling function, *M*(*x*, *y*, *z*), determines where the point measurements are taken in the phantom space. *h*(*x*, *y*, *z*) is the ultrasound system’s spatially dependant PSF, and will be discussed later in more detail. *u*(*x*, *y*, *z*) describes both the measurement noise as well as the physical phenomena which are not accounted for by the convolution model^11^. The noise model will now be discussed.

#### Noise Model

Scans from all coherent imaging modalities present with Speckle noise. This noise is a byproduct of the interfering echoes of a transmitted waveform that emanate from the studied object’s heterogeneities. Noise in ultrasound images is often modeled as:

2 $${g}_{e}(x,y,z)=f(x,y,z)\,{\xi }_{m}(x,y,z)+{\xi }_{a}(x,y,z)$$

where *g*_*e*_(*x*, *y*, *z*) is the observed envelope image, *f*(*x*, *y*, *z*) is the noise-free image, *ξ*_*m*_(*x*, *y*, *z*) is the multiplicative speckle noise component, and *ξ*_*a*_(*x*, *y*, *z*) is the additive speckle noise component^11^.

With ultrasound images, evidence exists that only the multiplicative noise needs to be considered^11^. Therefore, the additive noise term can be removed from (2) and *g*(*x*, *y*, *z*) can be expressed as:

3 $${g}_{e}(x,y,z)=f(x,y,z)\,{\xi }_{m}(x,y,z)$$

Taking the log of (3) would turn the multiplication into a simple addition problem:

4 $$\mathrm{log}\,({g}_{e}(x,y,z))=\,\mathrm{log}\,(f(x,y,z))+\,\mathrm{log}\,({\xi }_{m}(x,y,z\mathrm{)).}$$

There were a few distributions proposed to model speckle^11^, empirical tests done on the envelop data captured by the data acquisition unit show that the Generalized Gamma distribution has the best fit. The noise samples of the logarithmic transformed multiplicative noise in (4) can be modeled with the FisherTippett distribution given by:

5 $$p(I\,(x,y,z))=2\,\exp \,[\mathrm{(2}I\,(x,y,z)-\,\mathrm{ln}\,2{\sigma }^{2})-\exp \,\mathrm{[2}I\,(x,y,z)-\,\mathrm{ln}\,2{\sigma }^{2}]]$$

where *p* is the probability density function (PDF), *I*(*x*, *y*, *z*) denotes voxel intensity at point (*x*, *y*, *z*), and *σ* is their standard deviation.

The spatially varying PSF presented in (1), the sparsity due to the sampling function also presented in (1), and the noise model in (5) can all be incorporated into our random field based model. The details of their mathematical incorporation will be presented in the signal processing section.

#### PSF Characterization

The PSF characterization was done independently from the data acquisition unit, but was incorporated along with the acquired data as input to the signal processing unit. We will outline the model used to estimate the PSF, and then briefly discuss the origin of edge waves in the row-column method.

One of the most commonly used models for the point spread function of ultrasound systems is the one based on the Tupholme-Stephanisshen model for spatial impulse response, which was further derived for the pulse echo case by Jensen^12^. In this model, the point spread function of a row-column system at point ${\overrightarrow{r}}_{1}$ with transducers at point ${\overrightarrow{r}}_{2}$ and geometry S is given by:

6 $${H}_{pe}({\overrightarrow{r}}_{1},{\overrightarrow{r}}_{2},t)=h({\overrightarrow{r}}_{1},{\overrightarrow{r}}_{2},t)\ast h({\overrightarrow{r}}_{2},{\overrightarrow{r}}_{1},t)$$

where *δ*(.) is the Dirac delta function and *c* is the speed of sound at homogeneous medium of density *ρ*_0_, and *h*(${\overrightarrow{r}}_{1}$, ${\overrightarrow{r}}_{2}$, *t*) is the one way impulse response:

7 $$h({\overrightarrow{r}}_{1},{\overrightarrow{r}}_{2},t)={\int }_{S}\,\frac{\delta (t-\frac{|{\overrightarrow{r}}_{1}-{\overrightarrow{r}}_{2}|}{c})}{2\pi |{\overrightarrow{r}}_{1}-{\overrightarrow{r}}_{2}|}\,{\rm{d}}S$$

It should be noted that the convolution in (6) is over time and not space^13^.

This model is used in Field II, an ultrasound simulator for MATLAB, to estimate the PSF of various transducer setups. We will adopt this model when estimating the PSF in our framework. Given the intrinsic nature of row-column, it will be incorporated in the estimated PSF.

Acoustically, the row-column method is different from fully addressed 2-D arrays. Line elements are significantly longer than the length of the square line elements used in fully addressed 2-D arrays, which results in prominent edge artifacts. A study done in Rasmussen *et al*.^4^ showed that the two-way impulse response of a row-column system contains up to nine responses. Given nine echoes from a single scatterer, only the first echo can be used for imaging; the other ghost echoes are too weak, but they still degrade image quality.

The study suggests that electronic apodization is not an adequate form of apodization to solve the edge artifact problem, and propose that apodization should be integrated into the transducer array itself. This forms the basis for their integrated apodization row-column system.

In this work, since we are motivated by finding an image reconstruction framework that can addressed the problem of edge artifacts, we will be incorporating the field II model of the PSF into our signal processing unit.

## Signal Processing Unit

Figure 3 shows how the signal processing unit contributes to the overall system. This unit is the framework that drives the reconstruction of the ultrasound image. It incorporates the intrinsic properties of ultrasound as well as the acquired raw data into a MEG-SFCRF framework capable of addressing the challenges common to the row-column method. Raw data is mapped into a regular lattice and passed on to the optimization algorithm, once it converges the resulting image is displayed. The mathematical expression that drives this optimization will now be formulated.

### MEG-SFCRF Formulation

To estimate the tissue reflectivity function *f*(*x*, *y*, *z*), the inverse problem of (1) needs to be solved. The relationship between observed image and actual signal can be modeled as a conditional probability of true signal given the observation. We can formulate the reconstruction problem as a Maximum a Posteriori (MAP) estimation problem^14–16^, where a solution is obtained by maximizing the posterior distribution *P*(*F*|*G*):

8 $${F}^{\ast }=\mathop{argmax}\limits_{\overline{F}}\,\{P(F|G)\}$$

where *F* ^*^, $\bar{F}$, and *G* are the MAP solution, the possible results set, and the observation respectively.

Conditional random field (CRF) is a powerful discriminative modeling method, first proposed by Lafferty *et al*.^17^, that can directly model the conditional probability *P*(*F*|*G*) without specifying any prior model *P*(*F*) and relaxing the conditional independence assumption *P*(*G*|*F*)^18^. The CRF model can be expressed as:

9 $$P(F|G)=\frac{1}{Z(G)}\,\exp \,(-\psi (F,G))$$

where *Z* is the partition function and *ψ*(·) is the potential function^17–22^. The potential function *ψ*(·) is the combination of any arbitrary unary *ψ*_*u*_(·) and pairwise *ψ*_*p*_(·) potential functions:

10 $$\psi (F,G)=\sum _{i=1}^{n}\,{\psi }_{u}({f}_{i},G)+\sum _{c\in C}\,{\psi }_{p}({f}_{c},G)$$

where *C* is a set of a clique structure for each node.

While the CRF model considers node interactions in a small neighbourhood, fully connected conditional random fields (FCRF) addresses node interaction in the global scale^23^. However, FCRF requires huge computational cost. One way to reduce this computational cost has been proposed by Shafiee *et al*.^19^, where a stochastic clique was introduced. In this clique structure, the connection between nodes are determined in a stochastic manner.

Following the stochastically fully connected CRF (SFCRF) model proposed by Shafiee *et al*.^19^ where the clique structure for each node is based on various stochastic indicator functions, we propose an additional edge based stochastic indicator function (hence the term “edge-guided”) to better preserve edges in the reconstructed image.

Since each node *i* is connected to all other nodes, a set of neighbours for node *i* is defined by:

11 $$N(i)=\{j|j=1:n,j\ne i\}$$

where |*N*(*i*)| = *n* − 1. The clique structure *C* can be represented as the pairwise clique:

12 $$C={\{{C}_{p}(i)\}}_{i=1}^{n}$$

13 $${C}_{p}(i)=\{(i,j)|j\in N(i),{1}_{\{i,j\}}^{S}=1\}$$

where ${1}_{\{i,j\}}^{S}$ is the stochastic indicator neighbour function that defines whether two nodes can construct a clique. This function, in this research, is a combination of three probability distributions:

14 $${1}_{\{i,j\}}^{S}=\{\begin{array}{ll}1 & {P}_{i,j}^{s}\cdot {Q}_{i,j}^{d}\cdot {R}_{i,j}^{e}\ge \gamma \\ 0 & {\rm{otherwise}}\end{array}$$

${P}_{i,j}^{s}$ and ${Q}_{i,j}^{d}$ are the probability distributions that incorporate the spatial information and data relation among the states, and ${R}_{i,j}^{e}$ is the proposed probability distributions that incorporates edge information into ${1}_{\{i,j\}}^{S}$. *γ* determines how sparse the graph is. ${P}_{i,j}^{s}$ is defined as:

15 $${P}_{i,j}^{s}=\exp \,(-\frac{{({d}_{e}(i)-{d}_{e}(j))}^{2}}{2{\sigma }_{p}^{2}})$$

where *σ*_*p*_ is a control factor that determines how much this probability function contributes to the overall stochastic indicator neighbour function, and *d*_*e*_(*i*) is the Euclidean distance from node *i* and the center of the neighbourhood considered. ${Q}_{i,j}^{d}$ is defined as:

16 $${Q}_{i,j}^{d}=\exp \,(-\frac{{(I(i)-I(j))}^{2}}{2{\sigma }_{q}^{2}})$$

where *σ*_*q*_ is the weight that determines how much this probability function contributes to the overall stochastic indicator neighbour function, and *I*(*i*) is the pixel intensity at node *i*. Similarly, ${R}_{i,j}^{d}$ is defined as:

17 $${R}_{i,j}^{e}=\exp \,(-\frac{{(B(i)-B(j))}^{2}}{2{\sigma }_{r}^{2}})$$

where *σ*_*r*_ is the weight that determines how much this probability function contributes contributes to the overall stochastic indicator neighbour function, and *B*(*i*) is the edge value at node *i*.

**Figure 5:**
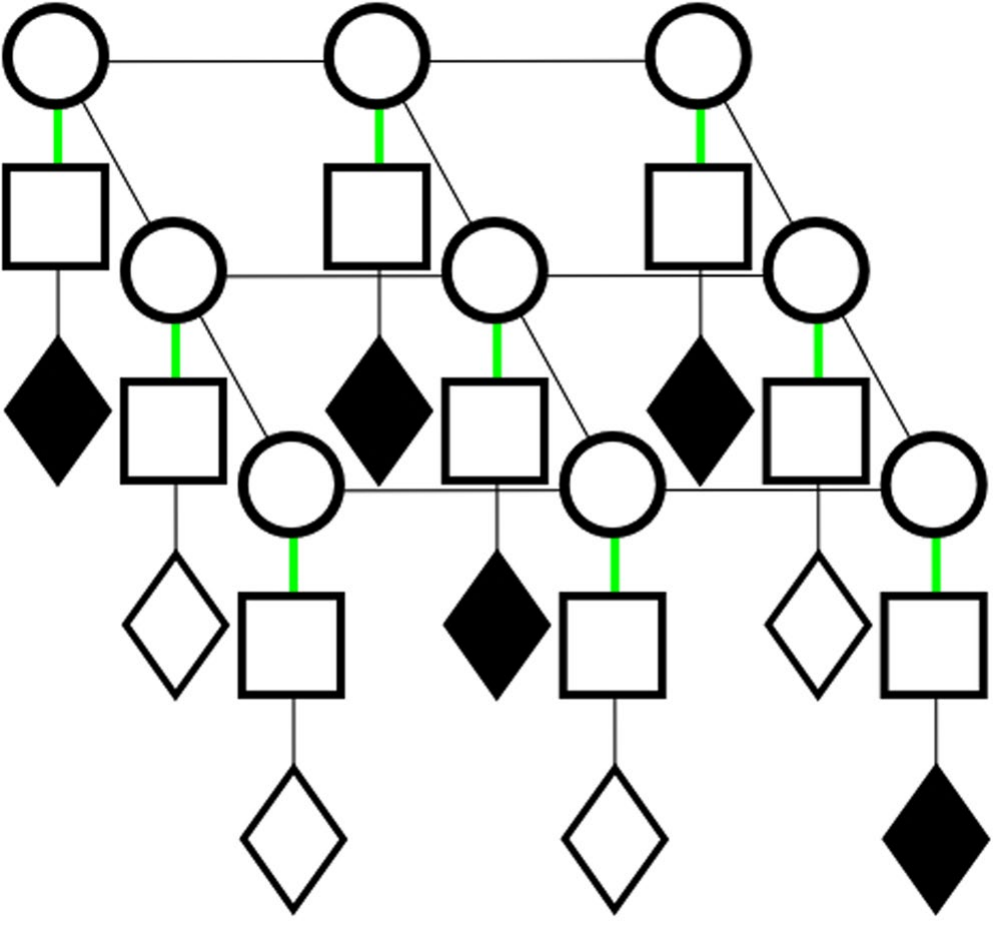
Visualization of the uncertainty layer within the state-observation model. This is a 2-D slice of the full 3-D lattice. The layers (from top to bottom) are state, observation, and uncertainty layer.

Regular CRFs adopt local cliques (or neighborhoods) where random variable interactions are involved in modeling. Neighbours are considered with the same degree of certainty in this model. Observations are assumed to be complete, and data sparsity is not taken into account^10^. However, one of the challenges this framework aims to address is to reconstruct a full 3-D volume *F* from a set of sparse measurements *G*. MCRF introduced an extension to the CRF model where a layer that determines the degree of the observation’s uncertainty is incorporated, thereby addressing the issue of incomplete data. With MCRF, every observation is linked with a value that specifies the uncertainty in modeling. With this extension, (9) can be rewritten as:

18 $$P(F|Cr,G)=\frac{1}{Z(G)}\,\exp \,(-\psi (F|Cr,G))$$

where *Cr* is the model’s uncertainty layer. *Cr* is a zero-one plane where *Cr* = 1 at positions with missing observations and *Cr* = 0 at positions where observations are available. Figure 5 demonstrates this layer in context with states and observations (where missing observations are black in this layer). This layer must be taken into account when the unary and pairwise functions are chosen.

The unary potential function plays the role of data-driven procedure, incorporating the information corresponding to the observation into the model. Since we believe that the observation is degraded according to the distribution shown in (5), FisherTippett noise is assumed as the degradation process and is incorporated in to the model as the unary potential function:

19 $${\psi }_{u}({f}_{i},G,C{r}_{i})=\{\begin{array}{ll}{\rm{\Psi }}({f}_{i},G), & C{r}_{i}={\rm{0}}\\ 0 & C{r}_{i}={\rm{1}}\end{array}$$

where Ψ(*f*_*i*_, *G*) is expressed as:

20 $${\rm{\Psi }}\,({f}_{i},G)=\frac{1}{\sigma }\,\exp \,(-\alpha \,\frac{\mathrm{log}\,G-\,\mathrm{log}\,H\,({f}_{i})}{\sigma })\cdot \exp \,(-\frac{\mathrm{log}\,G-\,\mathrm{log}\,H\,({f}_{i})}{\sigma })$$

where H denotes the function taking factors related to the imaging system (such as the spatially dependant PSF, sensor noise, etc.) into account, and *α* is the coefficient that determines the contribution of the observed data inside the ‘beams of readings’. The expression for Ψ(*f*_*i*_, *G*) comes from the Generalized Extreme Value theorem, which simplifies to the FisherTippett PDF expressed by (5).

**Figure 6:**
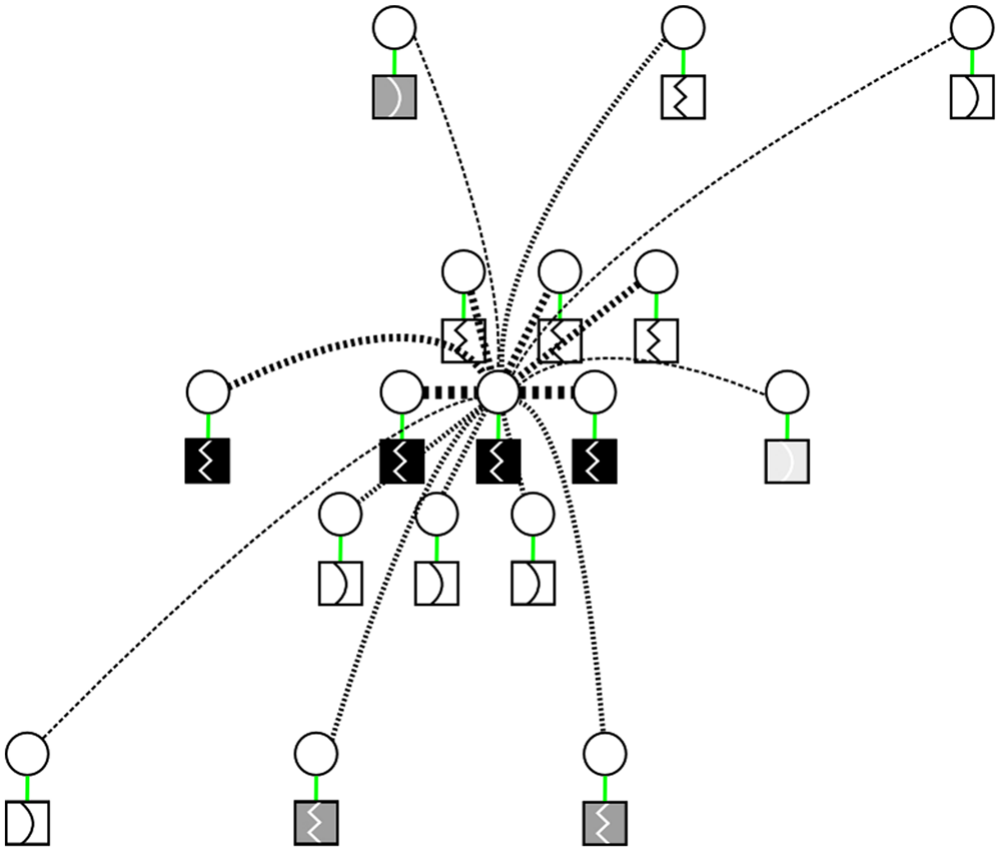
A realization of the pairwise relationship in the state-observation model. The different symbols inside the boxes indicate different edge values. The thickness of the dotted lines indicate how likely two nodes will be connected; each node in the graph will be connected according to a probability drawn from a distribution based on measurement, spatial location, and edge value. Nodes having similar measurement, spatial location, and edge are the most likely to be connected (thickest dotted lines), while nodes having no similarities are the least likely to form a connection (thinnest dotted lines).

The pairwise potential functions incorporates the spatial information into the model. These functions are defined based on a subset of random variables which is determined by clique structures. This is demonstrated in Fig. 6, where according to a predefined penalty function *w*(·), the relations among random variables in a clique *c* can be defined as:

21 $${\psi }_{p}({f}_{c},G)=\exp \,(-\beta |\,{f}_{i}-{f}_{j}|\cdot w({g}_{i},{g}_{j}))$$

where {*i*, *j*} ∈ *c*, *β* is the coefficient that determines the contribution of the spatial information, and *w*(*g*_*i*_, *g*_*j*_) is the penalty function. Note that *c* is simple clique, not to be confused with the uncertainty layer *Cr*.

The pairwise term aims to remove small noises, provide consistent labels in neighboring random variables and estimate the areas of the image with no prior data with the help of penalty functions based on the spatial information available. The penalty function attempts to use whatever information that is already available to find the best estimate for the ‘dark’ areas of the image. For the penalty function, two penalty terms are included: spatial proximity penalty term *w*_*sp*_ and First Order Variation (FOV) of intensity values *w*_*fov*_.

*The spatial proximity penalty term* is based on the assumption that the farther a voxel is, the less likely it is to belong to a unique segment of an image. It maintains the homogeneity of surrounding voxels. The spatial proximity between voxels *i* and *j* is quantified by the Euclidean distance *d*_*E*_(*i*, *j*):

22 $${w}_{sp}(i,j)=\exp \,(\frac{-{d}_{E}(i,j)}{2{\sigma }_{sp}^{2}})$$

where *σ*_*sp*_ is a control factor used to enforce the strength of spatial closeness.

*The first order variation* (*FOV*) *penalty term* is built on the need to preserve the boundaries of the estimated image, it uses the difference in intensities between neighbouring voxels to outline tissue transitions and provide a more clear ultrasound image. The penalty term is expressed as:

23 $${w}_{fov}({g}_{i},{g}_{j})=\exp \,(-\frac{\Vert {g}_{i}-{g}_{j}\Vert }{2{\sigma }_{fov}^{2}})$$

where *σ*_*fov*_ is a control factor used to enforce the strength of this penalty term.

Given these two penalty terms and the fact that stochastically fully connected random fields model longer range inter-node connections, this framework should avoid the excessive smoothing of inhomogeneous areas and boundaries^19, 24^. The data driven stochastic indicator function (16) strengthens the maintenance of inhomogeneous areas and the edge driven stochastic indicator function (17) helps maintain edges by preventing oversmoothing.

### Energy Function Inference

Given the MEG-SFCRF expression in (18) together with the potential function in (10), the energy function for the MAP model can be formulated as:

24 $$E(F,G,Cr)=\sum _{i=1}^{n}\,{\psi }_{u}({f}_{i},G,C{r}_{i})+\sum _{c\in C}\,{\psi }_{p}({f}_{c},G\mathrm{).}$$

The MAP can now be reformulated as:

25 $${F}^{\ast }=\mathop{argmin}\limits_{\overline{F}}\,\{E(F,G,Cr)\}.$$

To solve this MAP problem, a gradient descent algorithm was used. Gradient descent is an iterative optimization algorithm that finds the minimum by taking steps that are proportional to the negative of the gradient at a certain point. The gradient descent for possible solution *F* ^*^ can be expressed as:

26 $${F}^{{\ast }^{t+1}}={F}^{{\ast }^{t}}+\frac{\nabla E(F,G,Cr)}{\nabla F}$$

where $\frac{\nabla E(F,G,Cr)}{\nabla F}$ is the energy gradient with respect to *F* and ${F}^{{\ast }^{t}}$ is the estimated solution at iteration *t*. To find the possible solution *F* ^*^ while taking into account the energy function given in (24) and the potential functions given in (19) and (21), the gradient descent in (26) can be rewritten as:

27 $${F}^{{\ast }^{t+1}}={F}^{{\ast }^{t}}+\alpha (\frac{\nabla {\psi }_{u}(F,G,Cr)}{\nabla F})+\beta (\frac{\nabla {\psi }_{p}(F,G)}{\nabla F})$$

where $\frac{\nabla {\psi }_{u}(F,G,Cr)}{\nabla F}$ is the gradient of the unary part of the energy function with respect to *F*, $\frac{\nabla {\psi }_{p}(F,G)}{\nabla F}$ is the gradient of the pairwise part of the energy function with respect to *F*, *α* determines the contribution of the unary part of the energy function, and *β* determines the contribution of the pairwise part of the energy function.

## Experimental Setup

To evaluate the efficacy of our proposed system, EG-CRCUIS was tested on both simulated and real ultrasound scans. Simulated scans were compared against CRC-UIS^10^, the real-time CMUT row-column system used in Chen *et al*.^6^, the integrated apodization system proposed in refs 4 and 7, all with 128 by 128 elements that are 4.8 mm by 0.12 mm in dimension, as well as a system with a fully-addressed 2-D array. Real scans were only compared against CRC-UIS and the real-time CMUT row-column system. Simulations were performed using Field II, an open source MATLAB toolkit that has been used in ultrasound literature^25^. For both simulated and real evaluations of EG-CRCUIS and CRC-UIS, RF-data was envelope-detected, log-compressed, and mapped into a regular 3-D lattice through linear interpolation before passing it to the optimization stage.

### Simulation

In this work, Field II was used for all simulation: generating phantom data, performing ultrasound beamforming, and calculating the PSF of the EG-CRCUIS and CRC-UIS systems at different depths.

For the simulation, all tested systems were implemented with 32 × 32 2-D row-column addressing (with the exception of the fully addressed 2-D array), and the center frequency was set to 6 MHz, F-number on receive was 4. No attenuation was applied.

To create the phantom data, a general scatterer based on the required phantom dimensions and positions was made. Amplitudes with a Gaussian distribution were randomly spaced inside the set scattering region. There were 500,000 total scatterers inside the set region to ensure we get fully developed speckle. The amplitudes inside the predefined cyst positions was set to ten times the amplitude outside. The x-y-z positions all amplitudes were recorded to be loaded later.

To generate the simulated data, the transducer apertures were first defined. Apertures for emission and reception were then generated, with the impulse response and excitation of the emit and receive aperture set. The x-y-z positions of all amplitudes recorded when the phantom was made was then loaded, where beamforming in a manner identical to real row-column imaging devices was performed by Field II.

To model the PSF at a particular depth, the transducer apertures were first defined. Apertures for emission and reception were then generated. A point phantom at the required depth was created, and a linear sweep was then made to calculate the response. A point scatterer was then generated and the PSF at the required spatial location was found.

### Simulated phantom

**Figure 7:**
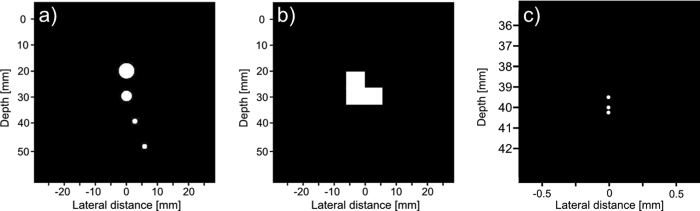
Model of simulated phantoms. The first phantom (**a**) consists of 4 cysts placed 10 mm apart in depth, with the third and fourth cysts placed 2.5 mm and 5 mm to the right respectively. The diameter of each cyst from top to bottom is 6 mm, 4 mm, 2 mm, and 2 mm. The second phantom (**b**) is a combination of three 6 mm by 6 mm squares that are arranged to form an “L” shape. The third phantom (**c**) is a series of point sources at [x, y, z] = (0, 0, 39.5) mm, (0, 0, 40) mm, and (0, 0, 40.25) mm.

Three phantoms, shown in Fig. 7, were used in the simulated tests. The first phantom consists of four cysts of decreasing diameter, each 10 mm farther away from the transducer. The bottom two cysts are placed 5 mm and 10 mm to the right of the center axis, this is to test our framework on objects that are off axis. The second phantom is a combination of three 6 mm by 6 mm squares arranged in an “L” shape. This is to test our phantom on a different homogeneous shape. The third phantom is a series of point sources placed at [x, y, z] = (0, 0, 39.5) mm, (0, 0, 40) mm, and (0, 0, 40.25) mm. This is to see how well our proposed system resolves close scatterers.

### Real Data

For the EG-CRCUIS, CRC-UIS, and the real-time CMUT row-column systems, the volumetric scanning data was acquired by a customized imaging system built using the PCI eXtensions for Instrumentation (PXI) platform. A row-column addressing capacitive micromachined ultrasonic transducers array (RC-CMUTs) was used, with full-width-half-max resolution of 1.2245 and side-lobe level under 40 dB. The 32 by 32 two-dimensional array has a center frequency of 5.9 MHz, an aperture size of 4.8 mm by 4.8 mm with a 150 pitch. Pre-amplifiers were used since CMUTs have small current output signals. The PXI system includes a 32 channel digitizer (NI-5752, National Instruments), a FPGA board (NI-7954, National Instruments), and an embedded controller module, which includes an Intel Core 2 Quad 2.26 GHz CPU and a Windows 7 operating system. An external FPGA was responsible for transmit beamforming while a set of high-voltage pulsers (LM96551, Texas Instruments) responsible for stepping the voltage to 30 V were used. The CMUTs were biased at −60 V to improve sensitivity and was operated in conventional mode. The system block diagram is shown in Fig. 3.

Receive beamforming is done following the line-beamforming method detailed in ref. 4. Hilbert’s transform is used to detect the envelope of the summed signal follow. The depth and angle, both azimuth and elevation, are then processed with the reconstruction framework.

### Real phantom

For the real row-column ultrasound scan, wire target imaging was performed on the same target used by ref. 10. Four wires, all 644 in diameter, were arranged in a way to allow for a scan of their cross sections.

### Metrics for comparison

For the purpose of our implementation, Peak Signal to Noise Ratio (PSNR), Effective Number of Looks (ENL), and Coefficient of Correlation (CoC) were used as metrics to evaluate the performance of our framework on simulated data. Contrast to Noise Ratio (CNR) and ENL metrics were used to evaluate the performance of our framework on real data. All metrics were defined according to recent literature^10, 26–32^.

PSNR is a metric that provides quality measure in terms of the power of the ideal and reconstructed image. As shown in (28), its is based on Mean Square Error (MSE) defined in (29). PSNR is frequently used in ultrasound noise despeckling literature to measure the performance of speckle removal^10, 26–32^. Higher PSNR indicate better image quality.

28 $$PSNR=10\,lo{g}_{10}\,(\frac{{(MAX({f}_{p}))}^{2}}{MSE})$$

where *f*_*p*_ is the ideal image, *MAX*(*f*_*p*_) is the peak signal of *f*_*p*_, and *MSE* is given by:

29 $$MSE=\frac{1}{MN}\,\sum _{i=1}^{M}\,\sum _{j=1}^{N}\,{({f}_{p,ij}-{f}_{r,ij})}^{2}$$

where *f*_*r*_ is the reconstructed image.

CoC is a metric that gives a measure of edge preservation. For completely uncorrelated images its value is 0, and for identical images its value is 1. Equation 30 shows the mathematical expression for CoC.

30 $$CoC=\frac{{\sum }_{i=1}^{M}{\sum }_{j=1}^{N}({\nabla }^{2}{f}_{p,ij}-{\overline{{\nabla }^{2}f}}_{p})\,({\nabla }^{2}{f}_{r,ij}-{\overline{{\nabla }^{2}f}}_{r})}{\sqrt{{\sum }_{i=1}^{M}{\sum }_{j=1}^{N}{({\nabla }^{2}{f}_{p,ij}-{\overline{{\nabla }^{2}f}}_{p})}^{2}\,{\sum }_{i=1}^{M}{\sum }_{j=1}^{N}{({\nabla }^{2}{f}_{r,ij}-{\overline{{\nabla }^{2}f}}_{r})}^{2}}}$$

where ${\nabla }^{2}$ is the laplacian operator $\overline{f}$ is the sample mean:

31 $$\overline{f}=\frac{1}{MN}\,\sum _{i=1}^{M}\,\sum _{j=1}^{N}\,{f}_{ij}.$$

ENL provides a measure of the statistical fluctuations (often introduced by speckle) in a particular region of interest; it gives an idea on how smooth a homogeneous region is. Given that we know what the image is supposed to look like, and we are working on homogeneous phantoms, smoothness can give an indication of how well we are reconstructing the homogenous regions as well as how well we are removing speckle from those regions. Higher ENL values indicate smoother regions. The mathematical expression for ENL is shown in (32), the ENL value is based on voxel mean *μ*_*t*_ and standard deviation *σ*_*t*_ of the region of interest *t*.

32 $$ENL=\frac{{\mu }_{t}^{2}}{{\sigma }_{t}^{2}}\mathrm{.}$$

CNR measures the difference between an area of an image feature and an area of background noise. Higher values indicate less noisey images. In the expression for CNR, *μ*_*b*_ and *σ*_*b*_ represent the mean and standard deviation of background noise, and *μ*_*r*_ and *σ*_*r*_ represent the mean and standard deviation of features of interest.

33 $$CNR=\frac{1}{R}\,(\frac{{\sum }_{r=1}^{R}({\mu }_{r}-{\mu }_{b})}{\sqrt{{\sigma }_{r}^{2}+{\sigma }_{b}}}).$$

## Results

To evaluate the performance of our proposed MEG-SFCRF reconstruction framework, the simulated output images from the EG-CRCUIS system were compared against simulated output images from the CRC-UIS^10^, the real-time CMUT row-column system by ref. 6, the integrated apodization system^4, 7^, and a system with a fully-addressed 2-D array implemented by us. The real image from the EG-CRCUIS system was compared against the CRC-UIS and the real-time CMUT row-column systems. The comparison was done both quantitatively as well as visually.

### Quantitative Evaluation

To quantify the performance of our reconstruction framework, metrics defined in recent related studies^10, 26–32^ were used. For the simulated data, comparisons were made between the output image and the ideal image; the original phantom image. For the real data, the metrics chosen account for the absence of ground truth.

The results of the EG-CRCUIS reconstruction were compared against the output of other systems in literature with the ideal phantom image as reference. Tables 1, 2 and 3 summarize the results for simulated first, second, and third phantom respectively. Table 4 summarizes the results of the real phantom.

**Table 1:** Quantitative results for the first simulated phantom.

System	PSNR (dB)	CoC	ENL
EG-CRCUIS	22.3878	0.2157	12.4943
CRC-UIS^10^	21.0310	0.1474	14.7624
real-time CMUT^6^	12.0393	0.0076	7.2600
Integrated apodization^8^	7.9748	0.0095	11.0534
Fully addressed 2-D array	19.8901	0.1872	0.6250

This table details the quantitative analysis of the simulated data for the first phantom on various systems from literature. Highest values are shown in bold.

**Table 2:** Quantitative results for the second simulated phantom.

System	PSNR (dB)	CoC	ENL
EG-CRCUIS	18.0949	0.1927	12.0111
CRC-UIS^10^	16.1575	0.1329	68.0186
real-time CMUT^6^	14.3155	0.1333	1.9662
Integrated apodization^8^	14.2250	0.1484	1.6230
Fully addressed 2-D array	16.4432	0.1587	5.7942

This table details the quantitative analysis of the simulated data for the second phantom on various systems from literature. Highest values are shown in bold.

**Table 3:** Quantitative results for the third simulated phantom.

System	PSNR (dB)	CoC	ENL
EG-CRCUIS	29.5291	0.5244	0.0815
CRC-UIS^10^	23.2665	0.2003	0.0465
real-time CMUT^6^	18.0784	0.0829	0.0898
Integrated apodization^8^	16.2192	0.0732	0.1292
Fully addressed 2-D array	19.4130	0.0142	0.1322

This table details the quantitative analysis of the simulated data for the second phantom on various systems from literature. Highest values are shown in bold.

**Table 4:** Quantitative results for the real phantom.

System	CNR (dB)	ENL
EG-CRCUIS	2.6510	49.0017
CRC-UIS^10^	1.5419	50.3531
real-time CMUT^6^	0.7703	23.0397

This table details the quantitative analysis of the real data. Highest values are shown in bold.

**Figure 8:**
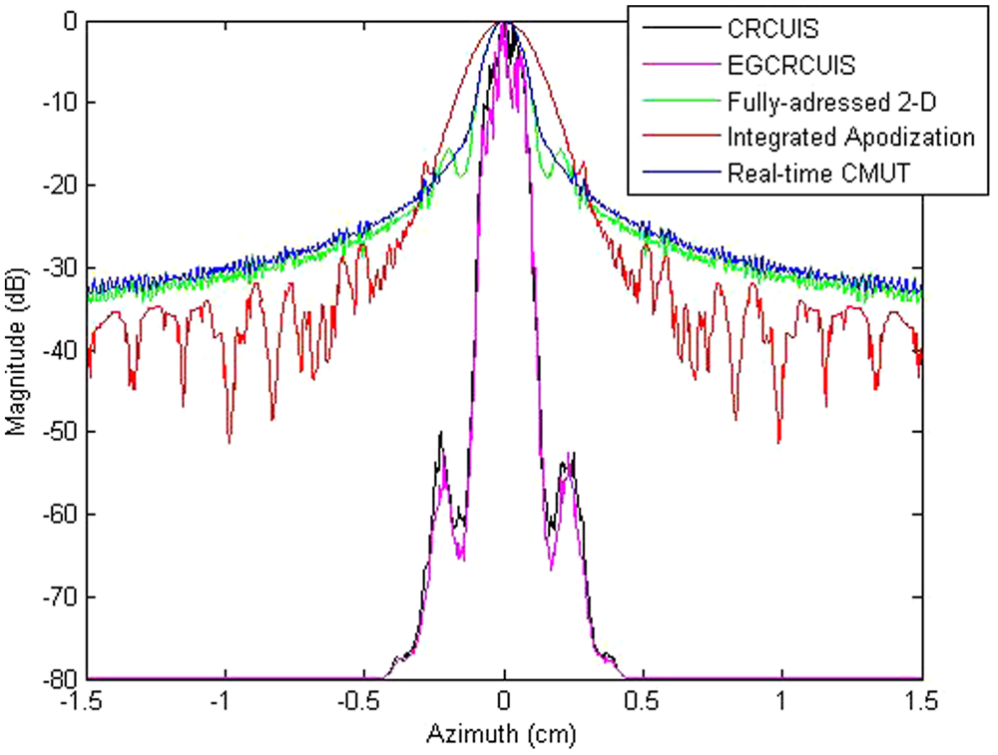
Beamplots derived from the PSFs of the different systems.

Figure 8 shows beamplots derived from the PSFs of all systems to outline the PSF and sidelobe level difference between the simulated systems. Both CRC-UIS and EG-CRCUIS have a narrower profile with lower sidelobe levels, with EG-CRCUIS having slightly lower profile. The main lobe of both EG-CRCUIS and CRC-UIS is not as smooth as the other systems, and there seems to be an imbalance between the right and left side starting at −20 dB, with the left side of the main lobes of both systems lower than the right side and with EG-CRCUIS slightly lower overall. Quantitative analysis of the resulting images based on the simultaed data shows that the proposed EG-CRCUIS system is capable of boosting its performance across PSNR and CoC while reducing ENL when compared to CRC-UIS. The increase in PSNR shows an improvement in noise reduction, and the increase in CoC shows an improvement in edge preservation. The reduction in ENL indicates that the EG-CRCUIS does not oversmooth the image as much as the CRC-UIS does. All three metrics for the EG-CRCUIS are higher than the other systems in literature.

For the real tests, the full-width-half-max resolution for the real-time CMUT, CRC-UIS, and EG-CRCUIS was found to be 1.2245 mm, 1.2557 mm, and 0.9292 mm respectively. Quantitative analysis of the images based on the real data shows that the EG-CRCUIS scored higher CNR than CRC-UIS, indicating better noise suppression. The ENL score for both EG-CRCUIS and CRC-UIS are very similar, although CRC-UIS is slightly higher. EG-CRCUIS outperforms the real-time CMUT system across both metrics.

### Visual Evaluation

**Figure 9:**
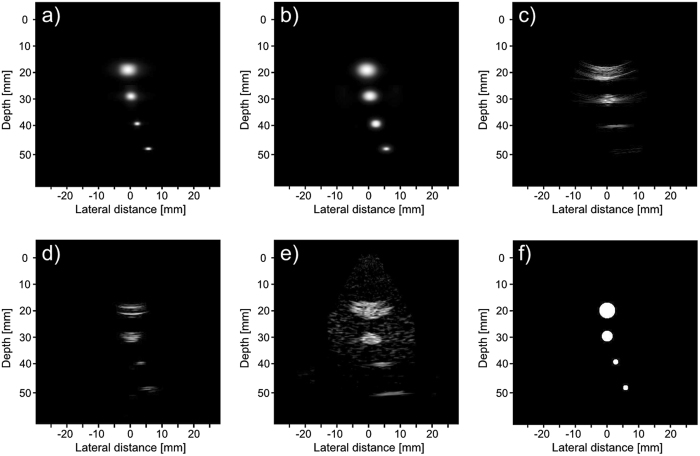
First phantom visual assessment of the EG-CRCUIS (top left) as opposed to other systems in literature. The EG-CRCUIS reconstruction is shown in (**a**), the CRC-UIS reconstruction^10^ is shown in (**b**), real-time CMUT system^6^ shown in (**c**), integrated apodization system^8^ shown in (**d**), fully addressed 2-D array shown in (**e**), and the original phantom image shown in (**f**). All simulated scans are shown at a dynamic range of 40 dB.

**Figure 10:**
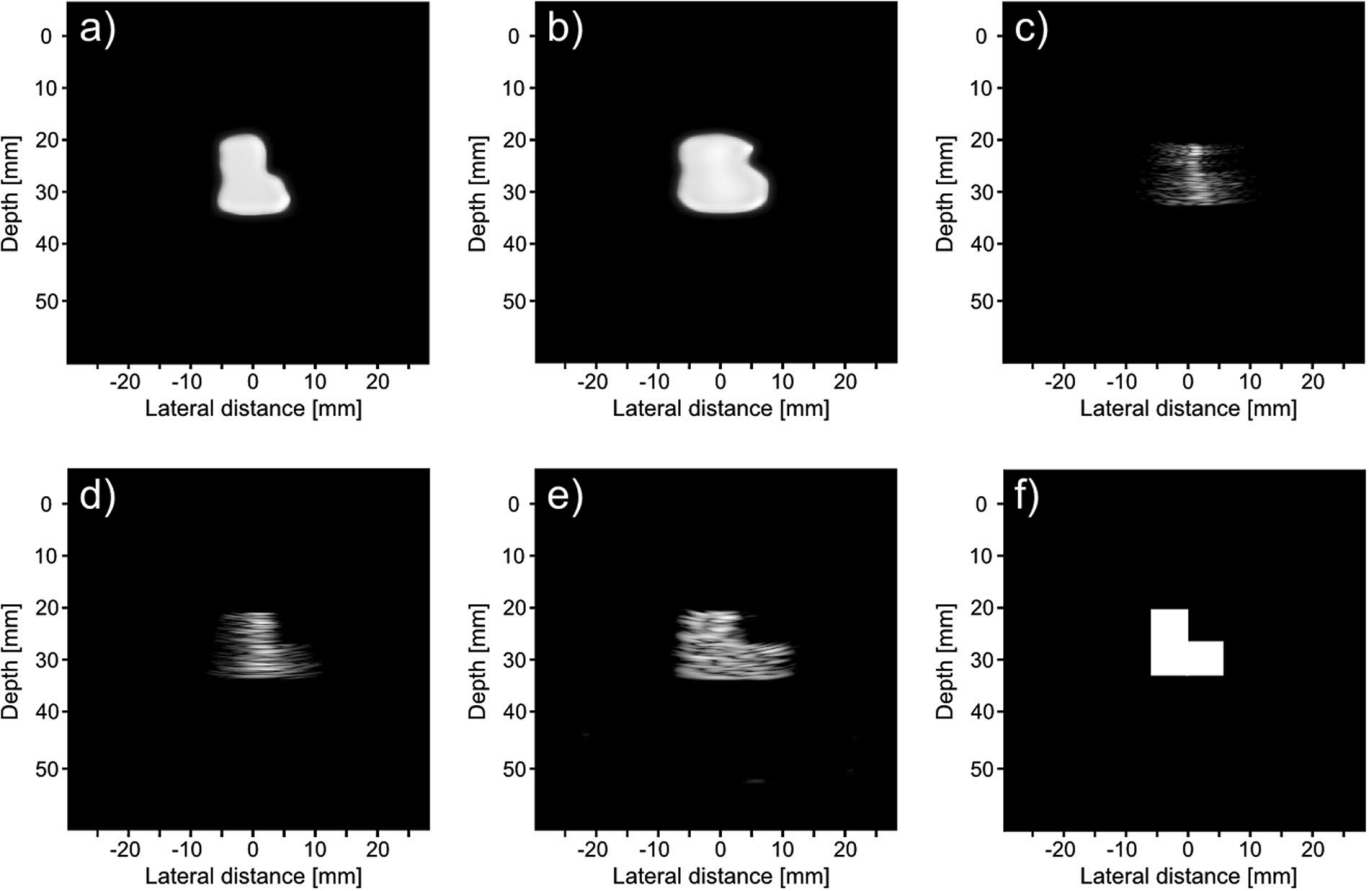
Second phantom visual assessment of the EG-CRCUIS (top left) as opposed to other systems in literature. The EG-CRCUIS reconstruction is shown in (**a**), the CRC-UIS reconstruction^10^ is shown in (**b**), real-time CMUT system^6^ shown in (**c**), integrated apodization system^8^ shown in (**d**), fully addressed 2-D array shown in (**e**), and the original phantom image shown in (**f**). All simulated scans are shown at a dynamic range of 40 dB.

**Figure 11:**
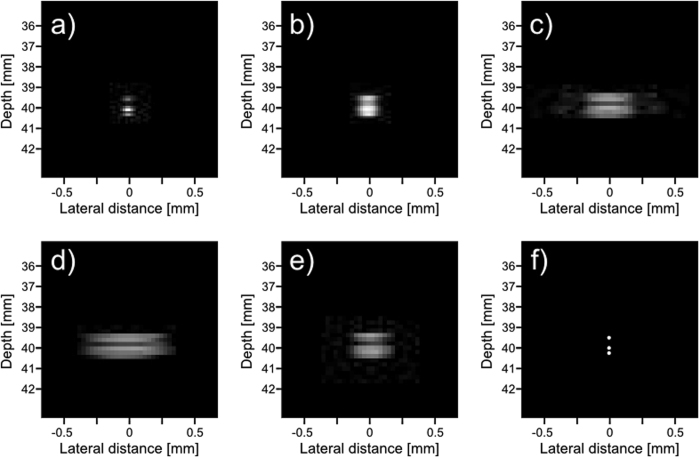
Third phantom visual assessment of the EG-CRCUIS (top left) as opposed to other systems in literature. The EG-CRCUIS reconstruction is shown in (**a**), the CRC-UIS reconstruction^10^ is shown in (**b**), real-time CMUT system^6^ shown in (**c**), integrated apodization system^8^ shown in (**d**), fully addressed 2-D array shown in (**e**), and the original phantom image shown in (**f**). All simulated scans are shown at a dynamic range of 30 dB.

Figures 9, 10 and 11 show the reconstruction of the first, second, and third simulated phantom respectively for the EG-CRCUIS and CRC-UIS as well as other systems in literature. Visual assessment with simulated phantoms shows that EG-CRCUIS presents images with less noise and more preserved edges when compared to the CRC-UIS and other systems in literature. These observations are also supported by the quantitative evaluation.

In the first simulated phantom images, the EG-CRCUIS shows more solid edges than the CRC-UIS, with cysts that are closer to the phantom image in terms of shape and size. Both systems also showed the best noise suppression when compared with other systems. The fully addressed 2-D system shows well sized cysts, but the image is noisy and the cysts are not smooth. The real-time CMUT system^6^ shows a lot of ringing artifacts where the cysts should be, and the shapes are not really clear. The integrated apodization system shows better suppression of ringing artifacts and clearer cysts than the real-time CMUT, however, it is not comparable to the other systems. The farthest point target is week for the real-time CMUT and the integrated apodization systems due to the fact that in simulation we used one 1-D array for transmit and one orthogonal 1-D array for receive, meaning the farthest point is slightly off axis. EG-CRCUIS and CRC-UIS were both able to reconstruct this better.

**Figure 12:**
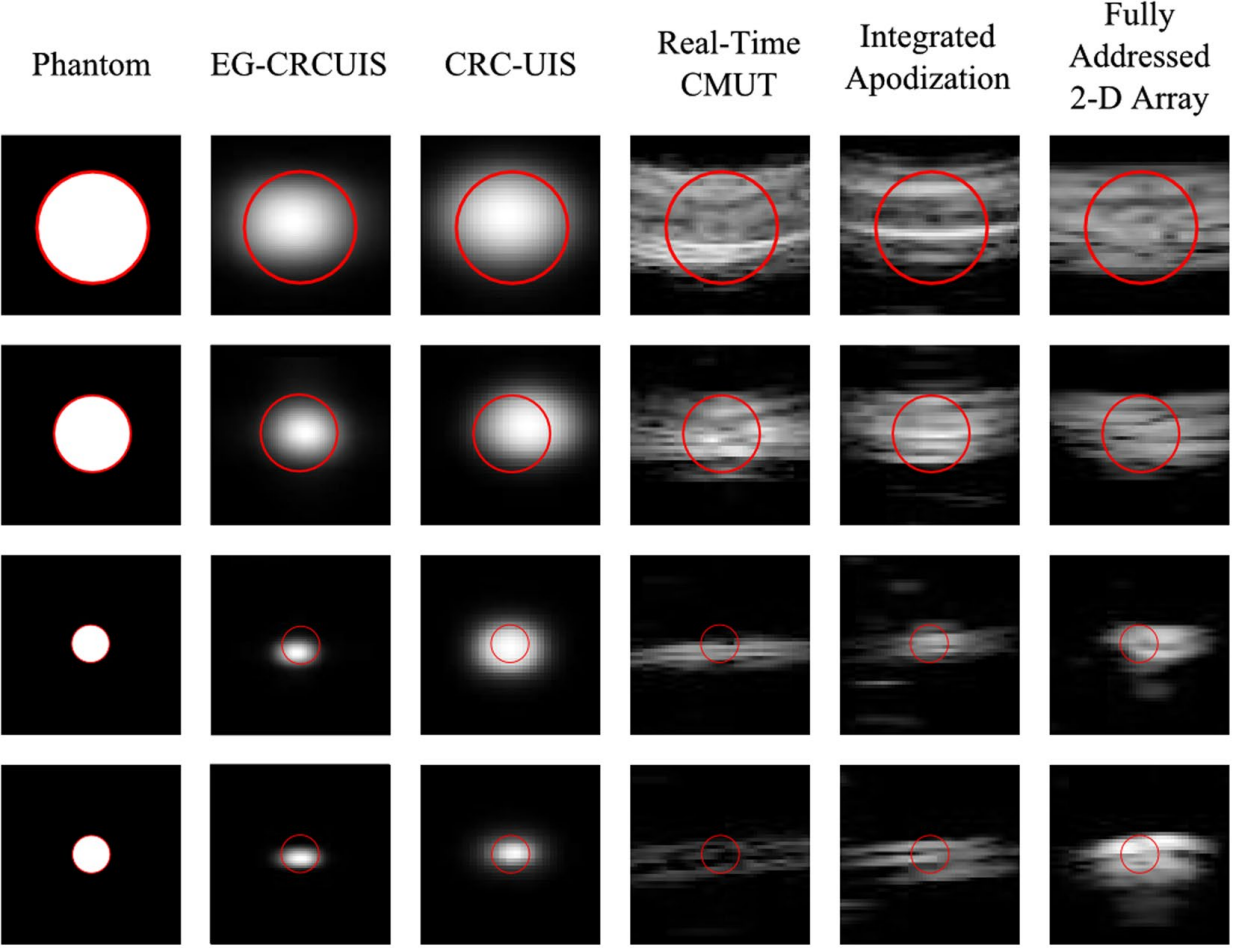
A closer look at the reconstruction of each cysts is shown. The red outline indicates the shape and position of the cysts in the phantom image. 30 dB is the dynamic range.

A closer look at the image reconstruction of the first simulated phantom of all systems is shown in Fig. 12. A slight right shift is seen in the second cysts is seen for EG-CRCUIS and CRC-UIS images and the third cyst in the fully addressed array. A slight downshift is seen in the third and fourth cysts of EG-CRCUIS as well as the third cyst for both CRC-UIS and the real-time CMUT.

In the second simulated phantom images, the EG-CRCUIS reconstruction is much closer in shape to the phantom when compared to CRC-UIS. EG-CRCUIS also shows edges more clearly than the real-time CMUT and the integrated apodization system. The fully addressed array shows the best shape reconstruction.

In the third simulated phantom images, only EG-CRCUIS was able to clearly resolve the bottom two point sources; in all other scans they were partially merged as one. In CRC-UIS, all three points merged into one, which highlights the tendency of the older system to oversmooth. The proposed system was able to resolve all three point sources, given that this system and its predecessor work on the same envelope data as the baseline RC one, this phantom strongly highlight the edge preservation capability this approach has.

**Figure 13:**
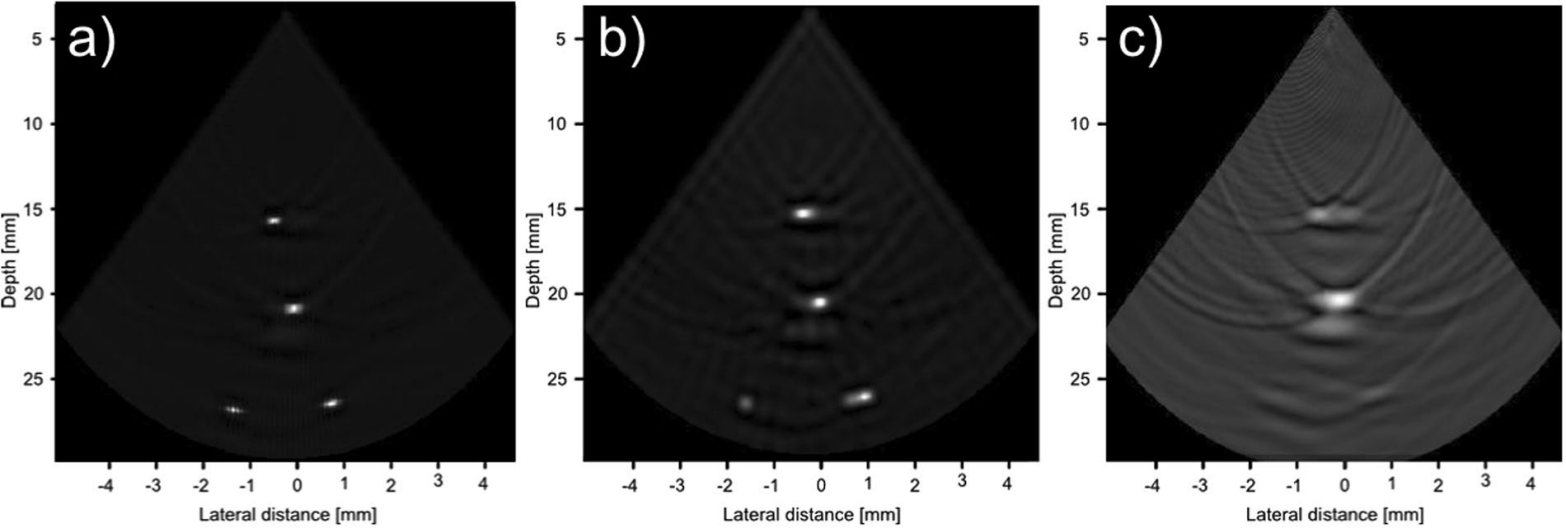
Visual assessment of the EG-CRCUIS (left side) as opposed to the CRC-UIS (middle) and the real-time CMUT system (right side). The EG-CRCUIS reconstruction shows better noise reduction, with the bottom two wires not visible with the real-time CMUT system. 40 dB is the dynamic range.

**Figure 14:**
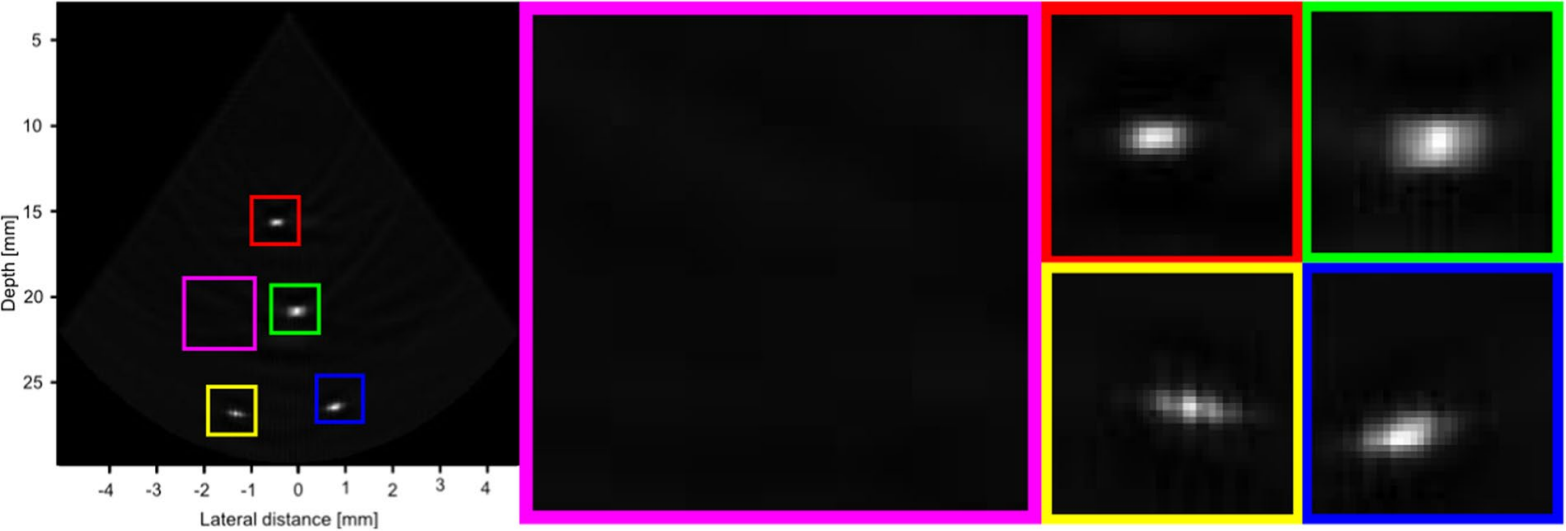
A closer look at the EG-CRCUIS reconstruction. Better noise suppression was achieved. 40 dB is the dynamic range.

**Figure 15:**
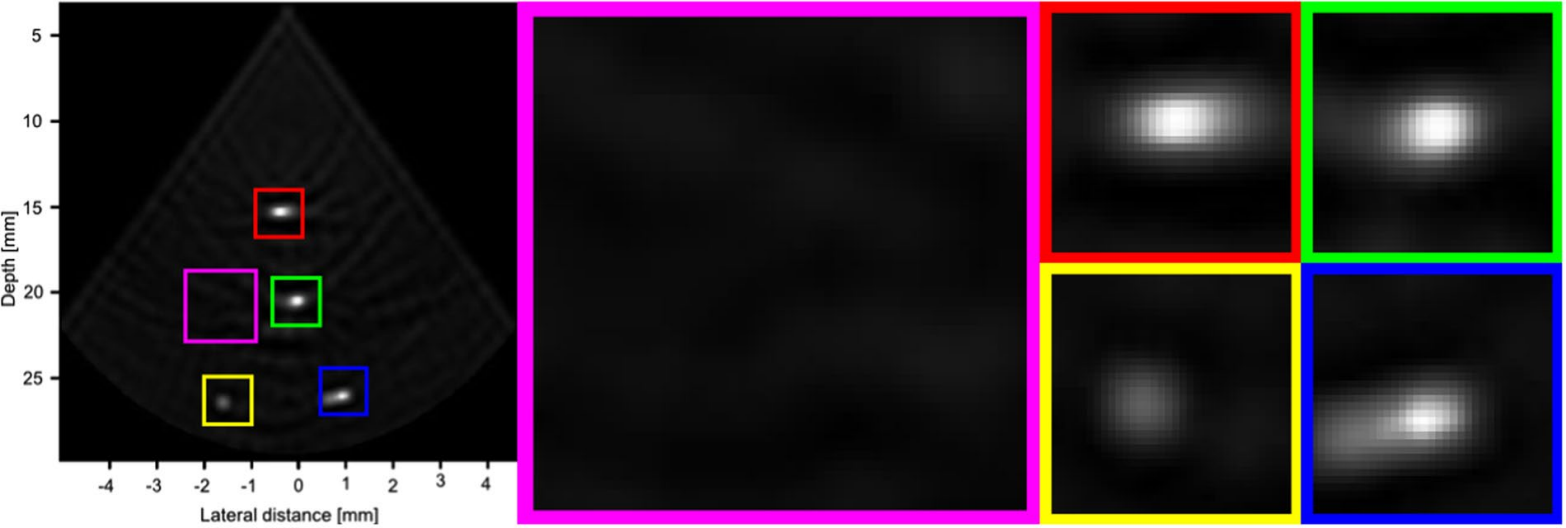
A closer look at the CRC-UIS reconstruction. Better noise suppression when compared with the real-time CMUT system, the bottom two wires can be seen. 40 dB is the dynamic range.

**Figure 16:**
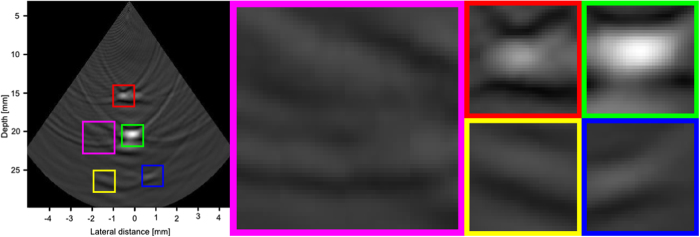
A closer look at the real-time CMUT system reconstruction. Very visible ringing artifacts can be seen. The bottom two wires cannot be seen, and the top wire is not very visible. 40 dB is the dynamic range.

Figure 13 shows the reconstruction of the real phantom data for both the EG-CRCUIS and CRC-UIS, as well as the real-time CMUT system. The EG-CRCUIS shows better noise suppression, and the bottom left wire is more clearly visible than the CRC-UIS. The real-time CMUT has only one clearly visible wire and has very noticeable ringing artifacts. A closer look at the image reconstruction of the EG-CRCUIS, CRC-UIS, and real-time CMUT is shown in Figs 14, 15 and 16 respectively. These observations are supported by the quantitative evaluation.

## Conclusion

### Summary and Discussion

In this research, we proposed EG-CRCUIS: an edge-guided compensated row-column ultrasound imaging system. The proposed system builds on CRC-UIS, a previously published row-column ultrasound imaging system. We introduced MEG-SFCRF: multilayered, edge-guided, stochastically fully connected random fields, a stochastic model that takes into account edge information as well as spatial and data relations when selecting a clique for the random field model. With this model that takes into account the underlying noise inherent to ultrasound as well as the missing data due to the sampling of the row-column method, and with the CRC-UIS’s ability to incorporate a the spatially varying, ghost artifact degraded PSF, we were able to better compensate for the row-column method’s inherent drawbacks. Through visual and quantitative evaluation, we were able to show that the proposed EG-CRCUIS system was capable of producing ultrasound images with more defined edges and less noise when compared to other systems in literature.

There are a few limitations to our method. First, we only use envelope data when reconstructing the image, which will not show objects that are close enough to interfere. Second, with the hardware used at the time of publication, real-time processing is not possible, and our proposed system is limited to applications with real-time image acquisition but no real-time feedback.

### Future Work

There are several directions to pursue in the future. First, a more comprehensive analysis on our image reconstruction framework for more bio-realistic/inhomogeneous targets needs to be done. Second, for the current study, the MEG-SFCRF reconstruction framework for CRC-UIS reconstructs each slice of the 3-D volume independently to form the final 3D image volume. Therefore, in the future we aim to extend the framework to adopt a full 3-D optimization in an efficient and effective way, which could have the potential for further improving image quality. Third, we aim to explore more comprehensive comparisons with other row-column imaging systems proposed in literature, which would necessitate the construction of these systems. Fourth, we will explore other random-field approaches for ultrasound image reconstruction.
